# Neutrophils negatively control IL-17A-producing γδ T cell frequencies in a contact-dependent manner under physiological conditions

**DOI:** 10.3389/fimmu.2025.1542191

**Published:** 2025-03-20

**Authors:** Xinhua Yu, Xiaoyang Yue, Junie D. Tchudjin Magatsin, Sebastian Marwitz, Jochen Behrends, Torsten Goldmann, Joseph T. Opferman, Brigitte Kasper, Frank Petersen

**Affiliations:** ^1^ Research Center Borstel, Leibniz Lung Center, Priority Area Chronic Lung Disease, Members of the German Center for Lung Research (DZLARCN), Borstel, Germany; ^2^ Key Laboratory of Basic Research on Regional Diseases, Education Department of Guangxi Zhuang Autonomous Region, College of Basic Medical Science, Guangxi Medical University, Nanning, Guangxi, China; ^3^ Histology, Research Center Borstel, Leibniz Lung Center, Borstel, Germany; ^4^ Airway Research Center North, Member of the German Center for Lung Research (DZL), Großhansdorf, Germany; ^5^ Core Facility Fluorescence Cytometry, Research Center Borstel, Leibniz Lung Center, Borstel, Germany; ^6^ Department of Cell and Molecular Biology, St. Jude Children’s Research Hospital, Memphis, TN, United States

**Keywords:** neutrophils, γδ T cells, IL-17A, cell death, physiological condition, innate immunity

## Abstract

**Background:**

In addition to serving as the primary effector cells against infections, neutrophils have been implicated in the regulation of both innate and adaptive immunity. In this study, we aimed to investigate the role of neutrophils in the regulation of the immune system under physiological conditions.

**Methods:**

The *in vivo* effect of neutrophils on the immune system was examined using neutropenic mice. The interaction between neutrophils and γδ T cells was investigated using an *in vitro* co-culture system.

**Findings:**

Unexpectedly, we observed an accumulation of γδ T cells in the cervical lymph nodes of neutropenic mice. Transcriptomic analysis revealed that these γδ T cells exhibited unique expression profiles of cell surface molecules and genes involved in defense responses. Further characterization indicated that the accumulated γδ T cells were IL-17 producing CD44^+^CD62L^−^CD27^−^ memory cells. Additionally, *in vitro* experiments demonstrated that neutrophils could inhibit the function of IL-17A producing γδ T cells by inducing cell death in a contact-dependent manner.

**Conclusion:**

This present study demonstrates that neutrophils negatively regulate IL-17 producing γδ T cells under physiological conditions. Given that IL-17A is a critical cytokine for the recruitment of neutrophils to peripheral tissues, our study suggests that the crosstalk between neutrophils and IL-17A producing γδ T cells is a crucial mechanism for maintaining immune homeostasis under physiological conditions.

## Introduction

Neutrophils are equipped with a multitude of toxic chemical weapons, establishing them as the primary effector cells in host defense against infectious organisms (1). Following maturation in the bone marrow, neutrophils migrate into the bloodstream. During acute inflammation, circulating neutrophils are recruited to peripheral tissues, where they become activated and combat pathogens (1). This recruitment can be initiated by innate immune cells, such as macrophages, mast cells, and γδ T cells, which sense danger signals (2). Additionally, adaptive immune cells or components, including Th17 cells and antibodies, can also trigger neutrophil recruitment (2). Consequently, neutrophils, despite their short lifespan, play a crucial role in acute inflammatory responses, primarily in pathogen clearance. Beyond their role in early host protection against invading bacteria and fungi, neutrophils have been shown to function as regulatory cells within both innate and adaptive immunoregulatory networks (3). Neutrophils engage in cross-talk with a variety of immune cell types, including dendritic cells (4), nature killer cells (5), γδ T cells (6) T and B cells (7, 8). Therefore, rather than a unidirectional communication, a bidirectional crosstalk between neutrophils and other immune cells appears to be crucial for immune system functionality. However, the relevance of such interactions *in vivo* under physiological conditions remains unclear, as most studies have been conducted *in vitro* or *in vivo* under pathological conditions (4–9).

In 2009, Steimer and colleagues reported a constitutive transgenic neutropenic LysM^Cre^-*Mcl*-1^-/-^ mouse strain (10). In this genetically modified mouse strain, *Mcl* gene is conditionally deleted within cells expressing high level of LysM gene, e.g. neutrophils. Consequently, LysM^Cre^-*Mcl*-1^-/-^ mice are deficient in matured neutrophils, providing an invaluable tool for investigating the effects of neutrophil deficiency. In this study, we utilized this neutropenic mouse strain to investigate the role of neutrophils in immune system regulation *in vivo* under physiological conditions by examining abnormalities in the immune system of these mice. Our results suggest a critical role of neutrophils in the regulation of IL-17 producing γδ T cells under physiological conditions.

## Materials and methods

### Mice

LysM^Cre^ mice were crossed to the Mcl-1^f/f^ line (both on C57BL/6-129 background) to generate neutropenic double transgenic *Mcl-1* ^f/f^ plus LysM^Cre^ mice (ho/tg) and its littermate control *Mcl-1* ^f/wt^ plus LysM^Cre^ (het/tg), as previously described (10). Animals were maintained in a specific pathogen-free housing facility, and received ovalbumin-free diet and water *ad libitum*. All experimental procedures were approved by the Animal Ethics Board of the Ministry of Environment, Kiel, Germany (Ref. 81-612).

### Flow cytometry

Single-cell suspensions were prepared from thymus, spleen and cervical LNs. To determine the cellularity of those lymph organs, cells were stained with FITC-rat anti-mouse CD3 (17A2, BD Bioscience, PerCP/Cy5.5 anti-mouse TCR γδ (GL3, Biolegend), PE-anti-mouse CD19 (6D5, Biolegend), PE/Cy7-anti-mouse CD4 (GK-1.5, Biolegend) and APC-anti-mouse CD8a (53-6.7, Biolegend). To characterized γδ T cells, cells were stained with FITC-rat Anti-mouse CD3, PerCP/Cy5.5 anti-mouse TCR γδ, PE-anti-mouse CD19 in combination with PE/Cy7-anti-mouse/rat/human CD27 (LG.3A10, Biolegend) or PE-Cy7-anti-mouse CD44 (IM7, Biolegend) and APC anti-mouse CD62L (MEL-14, Biolegend). To determine the IL-17A and IFN-γ expression in lymphocytes, cultured cells were stained with FITC-rat Anti-mouse CD3, PerCP/Cy5.5 anti-mouse TCR γδ, PE-anti-mouse CD19 and then intracellularlly stained with Alexa Fluor647-anti-mouse IL-17A (TC11-18H10.1, Biolegend) and PE/Cy7 anti-mouse IFN-γ (Clone: XMG1.2., Biolegend) Invitrogen™ LIVE/DEAD™ Viability/Cytotoxicity Kit (ThermoFisher Scientific) was utilized for all above staining to exclude dead cells. To detect the death of γδ T cells, cells were stained with APC-anti-mouse CD3, PerCP/Cy5.5 anti-mouse TCR γδ, FITC-annexin V (Bender MedSystems, Vienia, Austria) and propidium iodide (PI). Cells were detected using flow cytometric device BD™ LSRII (BD Bioscience). Data were analyzed with the FCSExpress software (DeNovo™ Software, Pasadena, CA, USA).

### Fluorescence-activated cell sorting

To purify γδ T cells, single-cell suspension was prepared from spleen and cervical LNs from mice. Cells were stained with PE-anti-mouse/human CD45R/B220 (RA-6B2, Biolegend), PE-anti-mouse TCR β chain (H57-597), PE-anti-mouse CD11c (N418, Biolegend), PE-anti-mouse CD49b (DX5, Biolegend) and PE-anti-mouse/human CD11b (M1/70) to exclude non-γδ T cells, with FITC-anti-mouse CD3 and APC-anti-mouse TCR γ/δ to define γδ T cells, and with PI to exclude dead cells. FACS sorting was performed by using BD FACS Aria™ IIu.

### *In vitro* differentiation of neutrophils

Neutrophils were differentiated *in vitro* from murine bone marrow (BM) cells. Briefly, BM cells were freshly isolated from wild-type mice and cultured at a concentration of 4×10^6^ cells/mL in complete RPMI medium supplemented with 5% fetal calf serum (FCS) and 20 pg/mL recombinant mouse granulocyte colony-stimulating factor (G-CSF) (R&D, 414-CS). The cultures were maintained at 37°C in a humidified incubator with 5% CO_2_ for 48 hours. Following incubation, dead cells were removed using Percoll density gradient centrifugation, and the remaining differentiated neutrophils were resuspended in fresh culture medium. The purity of the *in vitro*-differentiated neutrophils exceeded 95%, with a viability of 99%.

### Cell culture

Single-cell suspensions were prepared from cervical LNs for cell culture experiment to assess IL-17A and IFN-γ producing as well as γδ T cell viability. Cells were culture 2×10^6^ cells/mL in RPMI 1640 with 10 mM HEPES supplemented with 10% FCS, 2 mM L-glutamine, 100 U/mL penicillin, 100 μg/mL streptomycin, 0.05 mM ß-mercaptoethanol and 1 mM sodium pyruvate (all from PAN Biotech GmbH, Aidenbach, Germany). Additionally, 500 nM Brefeldin A was added, with or without 100 ng/ml PMA and 500 ng/ml Ca-Ionophor. *In vitro* differentiated neutrophils were co-cultured with γδ T cells at a 1:1 ratio for 5 hours. The transwell co-culture system was designed using transwell inserts with a poresize of 0.4 µm (Greiner Bio-One). Single-cell suspensions prepared from the cervical LNs were seeded into the wells of the 24-well plate, while neutrophils were seeded onto the transwell inserts.

To visualize the interaction between γδ T cells and neutrophils, γδ T cells sorted from cervical LNs and spleen of neutropenic mice were co-cultured with BM-derived wild-type neutrophils labeled using Vybrant cell labeling solutions (Molecular Probe, from Thomas). Cells were maintained in the previously described medium containing 500 nM Brefeldin A, 100 ng/ml phorbol myristate acetate n(PMA), 500 ng/ml calcium ionophore and 100 ng/ml DAPI. The cultures were prepared at a density of 4x10^6^ cells/ml, maintaining a γδ T cell-to-neutrophil ratio of 1:1. The cell suspension was seeded into a chamber and placed in a TCS SP5 II confocal microscope. The cells were cultured at 37°C for 200 minutes, during which a series of images were captured every 2.5 min using 63 x objective lens. These images were subsequently compiled to generate a time-lapse video to monitor γδ T cell–neutrophil interactions.

### RNA preparation and transcriptome analysis

Total RNA was isolated from 0.5 to 1 million purified living γδ T cells with the QIAmp Mini Kit (Qiagen) according to the manufacturer’s instructions. RNA integrity for microarray analysis was determined using the Agilent Bioanalyzer with the RNA Nano 6000 Kit according to the manufacturer’s instructions (Agilent). Transcriptome analysis was conducted with Agilent Mouse Gene Expression 4x44k V2 arrays. The cRNA were labeled and hybridized according the protocol we described previously (11), and scanned with an Agilent SureScan microarray scanner. Raw data was imported into GeneSpring version 13 with Percentile Shift as normalization method. Quality control was done on all samples and compromised probes removed from further analyses. To evaluate differentially expressed genes between groups, a Moderated T-Test was computed with a Fold-Change cut-off of 4 and a Benjamini-Hochberg multiple testing correction cut-off of p < 0.01. Unsupervised Hierarchical clustering of significantly expressed genes was done on log2-transformed, normalized intensity values of single samples according to Pearson Centered similarity measure. The enrichment analysis for the differentially expressed genes was performed with DAVID Bioinformatics Resources 6.8 software (12). Transcriptome data has been deposited at Gene Expression Omnibus (GSE272418) and will made freely available upon publication.

### Statistical analysis

All statistical analyses were meticulously conducted using GraphPad Prism Software. For the comparison between two groups, the Student’s t-test was utilized to assess statistical significance for normally distributed quantitative data, while the Mann–Whitney U test was employed for non-normally distributed data. For comparisons between multiple groups, a one-way ANOVA was used, followed by Tukey’s *post hoc* test for normally distributed data and Dunn’s *post hoc* test for non-normally distributed data. Probability values <0.05 were considered statistically significant.

## Results

### Accumulation of γδT cells in neutropenic mice

**Figure 1:**
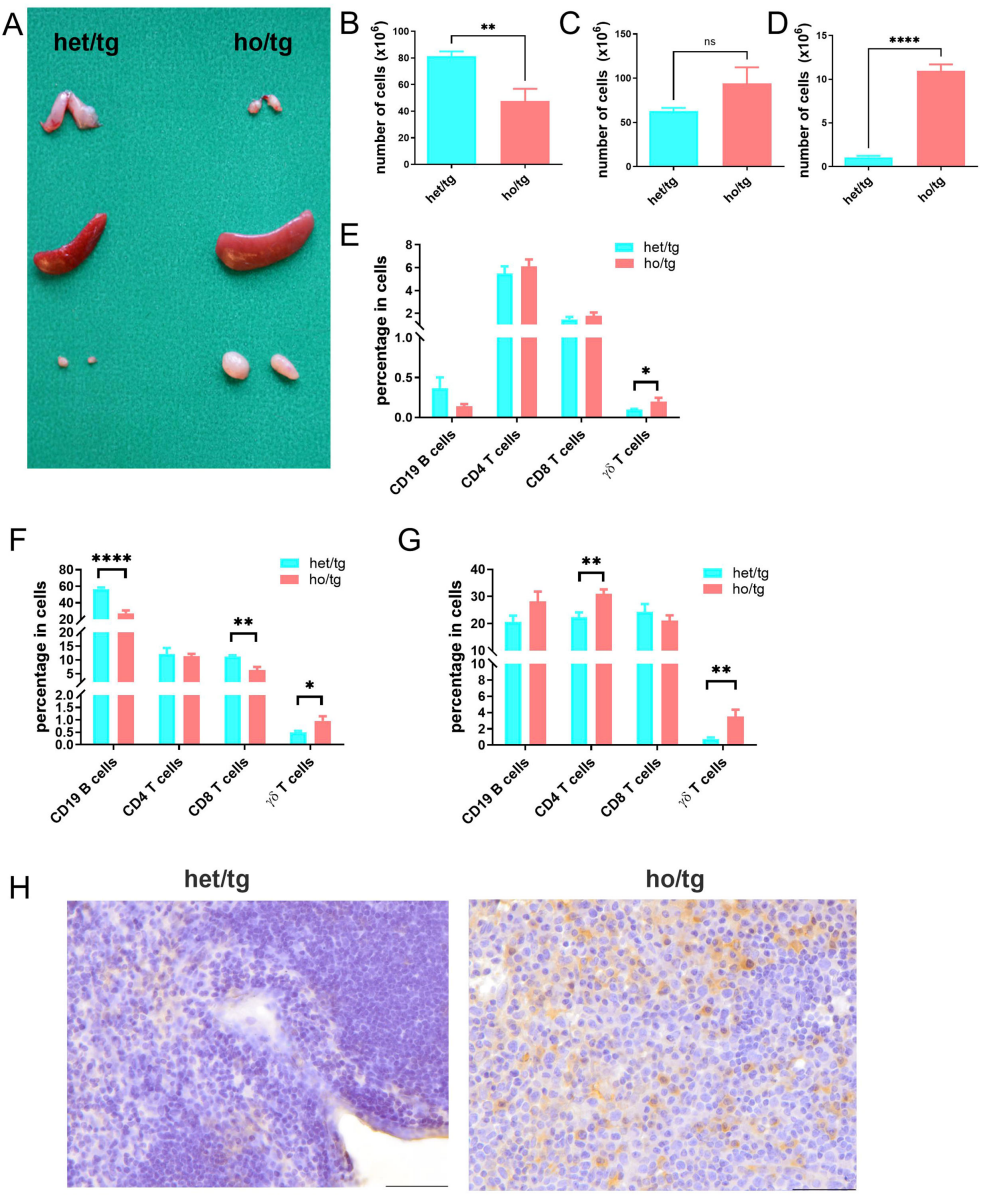
Accumulation of γδT cells in neutropenic mice. **(A)** Representative picture of thymus, spleen and cervical lymph nodes (LNs) from *Mcl-1* ^f/f^; LysM^Cre^ neutropenic male mice (ho/tg) and their littermate *Mcl-1* ^f/wt^; LysM^Cre^ (het/tg) control male mice at the age of 10-12 weeks old. Single cell suspensions were prepared from thymus, spleen and cervical LNs and counted and stained with anti-CD19, anti-CD4, anti-CD8, anti-CD3, and anti-γδTCR IgGs to defined B cells, CD4+ T cells, CD8+ T cells, and γδT cells. Total number of cells in thymus **(B)**, spleen **(C)** and cervical LNs **(D)** were compared between neutropenic mice (n=6) and control mice (n=6). Percentages of CD19+ B cells, CD4+ T cells, CD8+ T cells and γδT cells in thymus **(E)** spleen **(F)** and cervical LNs **(G)** were compared between neutropenic (n=6) and control (n=6) mice. **(H)** Representative picture of immunohistochemistry staining for γδT cell in cervial LNs of neutropenic and control mice. Bar=50 µm. Data are presented as mean ± SEM, ns, not significant; *p<0.05; **p<0.01; ****p<0.0001.

At 10-12 weeks of age, neutropenic mice were considerably smaller than their littermate controls in terms of both body size and body weight (18.2 ± 3.2 g vs 29.6 ± 2.7 g) (**Supplementary Figure 1**). To determine abnormalities in the immune system of neutropenic mice, we evaluated the thymus, spleen, and lymph nodes (LNs). Compared to their littermate controls, neutropenic mice exhibited a smaller thymus and a decreased number of thymocytes (**Figures 1A, B**; **Supplementary Figure 1C**), consistent with their reduced body weight. In contrast, the spleens of neutropenic mice were significantly larger than those of control mice, indicating splenomegaly, although the number of cells in the spleen was comparable between the two groups (**Figure 1C**; **Supplementary Figure 1D**). Moreover, the cervical LNs in neutropenic mice were approximately ten times larger in both weight and cell number than those in littermate controls (**Figure 1D**; **Supplementary Figure 1E**). Interestingly, aside from the cervical LNs, the lymphadenopathy was not observed in axillary, iliac, inguinal, popliteal or mesenteric LNs of neutropenic mice.

We further investigated the cellular composition of these lymphoid organs by determining the percentages of CD19^+^ B cells, CD4^+^ T cells, CD8^+^ cells and γδT cells (**Supplementary Figure 2**). Notably, the percentages of γδ T cells in the thymus, spleen, and cervical LNs of neutropenic mice were consistently higher than those in control mice (**Figures 1E–G**), whereas such differences were not observed in other lymphocyte populations. This suggests an accumulation of γδ T cells in neutropenic mice. Among the three lymphoid organs, the cervical LNs exhibited the highest degree of γδ T cell accumulation, followed by the spleen and then the thymus. The accumulation of γδ T cells in the cervical LNs was further confirmed by immunohistochemical staining (**Figure 1H**).

### Unique features of accumulated γδT cells in neutropenic mice

**Figure 2:**
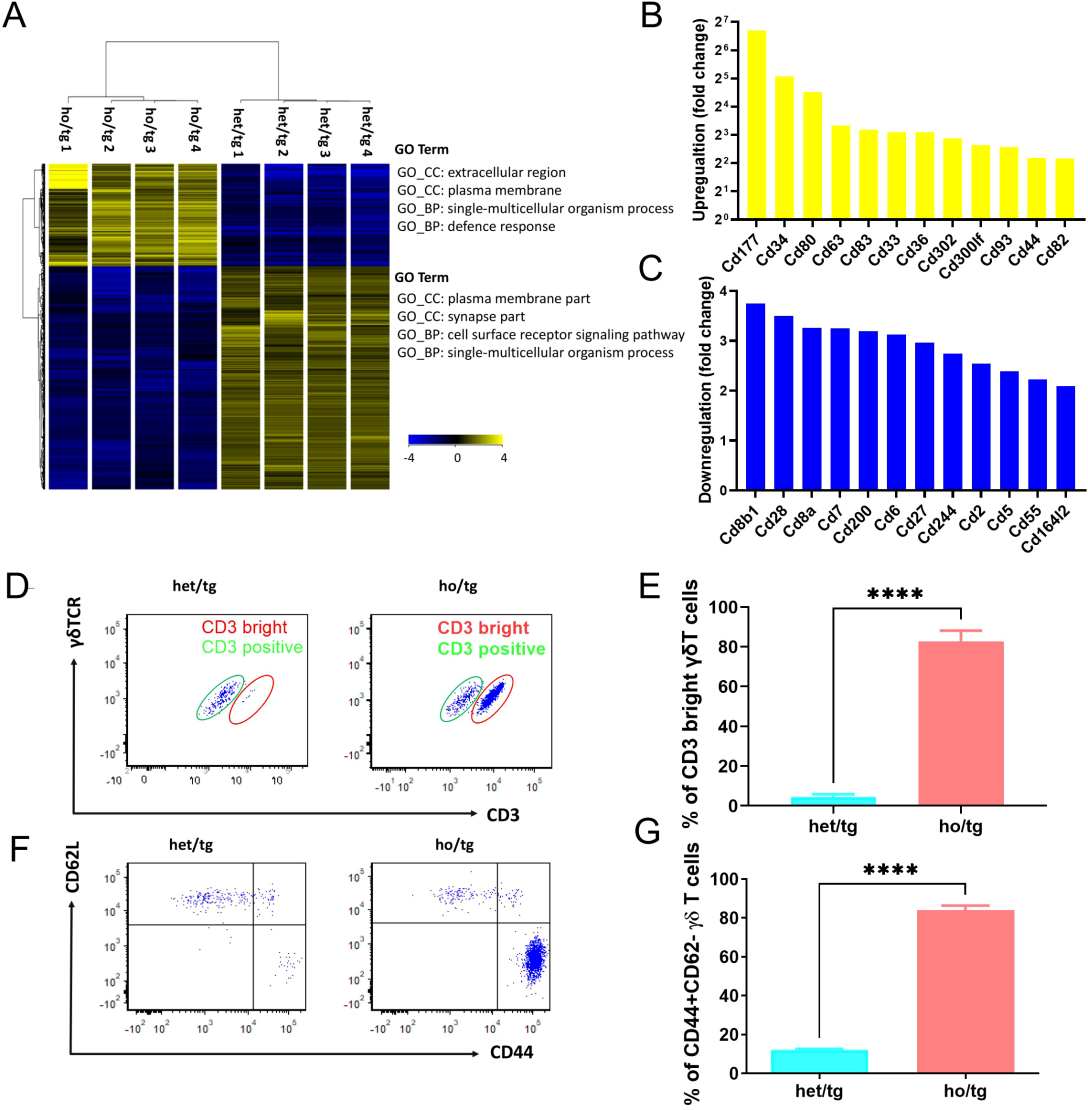
Unique features of γδT cells accumulated in neutropenic mice. Hierarchic cluster of differentially expressed genes between γδT cells from neutropenic and control mice. Blue color represents a low expression and yellow color stand for a high expression. GO term enrichment analysis was performed using DAVID 6.8 software for both upregulated and downregulated genes, and top enriched GO terms are indicated. Upregulated **(B)** and downregulated **(C)** gene encoding cluster of differentiation (CD) molecules. **(D-F)** Characterization of γδT cells isolated from neutropenic mice and littermate controls. Single cell suspension was prepared from cervical LNs from neutropenic and control mice characterized either by staining the freshly prepared cells with CD3, γδTCR, CD44 and CD62L. Expression of CD3 and γδTCR on γδT cells in neutropenic (n=5) and control (n=5) mice are shown as representative sample **(D)** and quantitative values of percentage of CD3 bright γδT cells **(E)**. Expression of CD62L and CD44 on γδT cells in neutropenic (n=5) and control (n=5) mice are shown as representative sample **(F)** and quantitative values of percentage of CD44+CD62L- γδT cells **(G)**. Data are presented as mean ± SEM, ****p<0.0001.

To characterize the accumulated γδT cells, we purified those cells from the spleen and cervical LNs of neutropenic and control mice using cell sorting. RNA was then isolated from these cells and subjected to transcriptome analysis. Principal component analysis of the samples revealed that γδ T cells from neutropenic mice differed significantly from those of control mice (**Supplementary Figure 3**). Even with stringent criteria (fold change >4 and corrected p <0.01), 3,040 genes were differentially expressed in γδ T cells from neutropenic mice compared to controls, including 959 upregulated genes and 2,081 downregulated genes (**Supplementary Table 1**; **Figure 2A**). **Table 1** summarizes the top 10 upregulated and downregulated genes between γδ T cells from neutropenic and wild-type controls. Notably, many neutrophil-related genes were among the top 10 upregulated genes, such as cathepsin G (*Ctsg*), myeloperoxidase (*Mpo*), proteinase 3 (*Prtn3*), and defensin alpha 2 (*Defa2*) and alpha 3 (*Defa2*). Two cytokine genes, interferon gamma (*Ifng*) and chemokine (C-C motif) receptor 10 (*Cxcl10*), were among the top 10 downregulated genes. Enrichment analysis revealed that the upregulated genes were enriched in Gene Ontology (GO) terms related to the extracellular region, plasma membrane, single-multicellular organism process and defense response. The downregulated genes were enriched in GO terms related to plasma membrane part, synapse part, cell surface receptor signaling pathway and single-multicellular organism process (**Supplementary Table 1**, **Figure 2A**). Thus, transcriptome analysis suggests that γδ T cells from neutropenic mice differ from controls in genes involved in plasma membrane and in cell functions, particularly in defense responses.

**Table 1:** List of top 10 upregulated and downregulated genes between γδT cells from neutropenic and littermate control mice.

	Gene	Description	Fold change	p (Corr)
**Upregulation**	*Ctsg*	cathepsin G	1269.8	1.02E-04
	*Mpo*	myeloperoxidase	1115.6	8.82E-05
	*Prtn3*	proteinase 3	809.5	1.19E-04
	*Defa2*	defensin, alpha, 2	535.4	1.41E-04
	*Ms4a3*	membrane-spanning 4-domains, subfamily A, member 3	522.8	6.71E-06
	*Mpo*	myeloperoxidase	491.0	2.59E-04
	*Gstm5*	glutathione S-transferase, mu 5	404.4	5.18E-05
	*Cebpe*	CCAAT/enhancer binding protein (C/EBP), epsilon	379.6	1.12E-05
	*Defa3*	defensin, alpha, 3	325.7	3.05E-04
	*Bicc1*	bicaudal C homolog 1	289.1	9.60E-06
**Downregulation**	*Rhox3a*	reproductive homeobox 3A	269.2	1.01E-04
	*Atf3*	activating transcription factor 3	46.0	3.34E-06
	*Ccr10*	chemokine (C-C motif) receptor 10	45.3	4.40E-04
	*AI481877*	expressed sequence AI481877	37.9	1.46E-04
	*Fam71a*	family with sequence similarity 71, member A	36.3	2.97E-06
	*Mal*	myelin and lymphocyte protein, T cell differentiation protein	35.6	7.21E-04
	*Ifng*	interferon gamma	33.4	3.64E-03
	*Nr4a2*	nuclear receptor subfamily 4, group A, member 2	33.3	2.47E-05
	*Zcchc12*	zinc finger, CCHC domain containing 12	32.8	6.18E-05
	*C1ql3*	C1q-like 3	30.2	3.54E-05

Given the significant differences in the expression of cell surface molecules between γδ T cells from neutropenic mice and their littermate controls, we focused on differentially expressed genes encoding cluster of differentiation (CD) molecules, which act as cell surface receptors or ligands. In total, 24 CD genes were differentially expressed in γδ T cells from neutropenic mice compared to controls, including 12 upregulated and 12 downregulated genes (**Figures 2B, C**). Among the upregulated genes, *Cd44* is of particular interest as it encodes CD44, a marker of memory T cells (13). To verify this, we characterized γδ T cells by staining cells isolated from the cervical lymph nodes (LNs) of neutropenic mice and their littermate controls with CD3, γδTCR, CD44, and CD62L. Notably, γδ T cells comprised two distinct populations: CD3^dim^ and CD3^bright^ γδT cells (**Figure 2D**). Approximately 95% of γδT cells isolated from wildtype mice were CD3^dim^ γδT cells. In sharp contrast, γδT cells from neutropenic mice comprised approximately 80% CD3^bright^ γδT cells (**Figure 2E**). The γδ T cells were further classified into subgroups using CD44 and CD62L to define naive cells (CD62L^+^CD44^-^), central memory cells (CD62L^+^CD44^+^), and effector memory cells (CD62L^-^CD44^+^) (14). As shown in **Figures 2F, G**, the percentage of CD62L^-^CD44^+^ γδT cells in the cervical LNs of neutropenic mice (84.1%) are drastically higher than that in control mice (12.1%) (*p*<0.0001), demonstrating that the accumulated γδT cells in neutropenic mice are effector memory cells.

### γδT cells in neutropenic mice are IL-17 producing cells

In the next step, we further characterized the accumulated effector memory γδ T cells in neutropenic mice by determining their function. Among the 12 downregulated genes encoding CD molecules, *Cd27* drew our attention, as it has been reported that CD27^-^ γδT cells are IL-17A producing γδT cells (15). At the transcriptome level, several IL-17A-associated genes, including *Il17a*, *Il17rc*, *Il17re*, and *Rorc*, were upregulated in γδ T cells from neutropenic mice compared to controls. Interestingly, two IL-17A-related genes, *Il17rb* and *Il17d*, were downregulated (**Table 2**).

**Table 2:** Differentially expressed genes encoding IL-17A-related molecules between γδT cells from neutropenic and littermate control mice.

Gene	Description	Regulation	Fold changes	p (Corr)
*Rorc*	RAR-related orphan receptor gamma	up	8.5	2.81E-04
*Il17a*	interleukin 17A	up	7.9	9.74E-03
*Il17rc*	interleukin 17 receptor C	up	5.2	4.42E-03
*Il17re*	interleukin 17 receptor E	up	4.8	3.23E-03
*Il17rb*	interleukin 17 receptor B	down	15.9	9.52E-04
*Il17d*	interleukin 17D	down	6.6	2.36E-04

**Figure 3:**
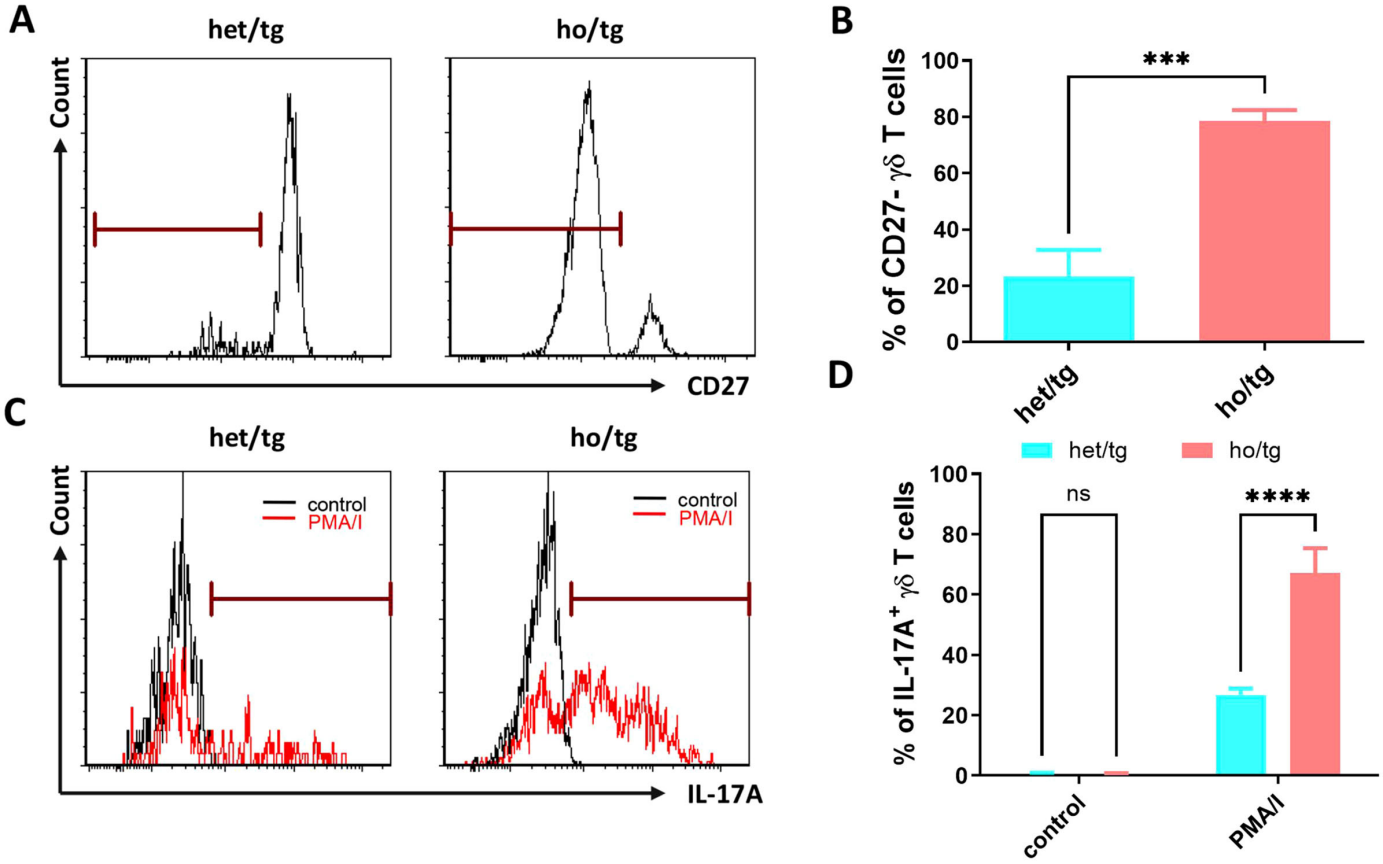
Production of IL-17A in γδT cells of neutropenic mice and littermate controls. Single-cell suspensions were prepared from cervical LNs from neutropenic and control mice characterized either by staining the freshly prepared cells with surface markers or by staining the cultured cells with anti-IL-17A IgG. Representative sample **(A)** and quantitative values of percentage of CD27- γδT cells **(B)** show the expression of CD27 on γδT cells in neutropenic (n=5) and control (n=5) mice. Freshly prepared cells from cervical LNs from neutropenic mice (n=6) and littermate controls (n=6) were cultured for 5 hours with/without stimulation of phorbol myristate acetate and calcium ionophore (PMA/I) and stained with anti-IL-17A IgG to determine the IL-17A production in γδT cells. Representative samples **(C)** and quantitative values **(D)** of percentage of IL-17A-producing γδT cells are shown. Data are presented as mean ± SEM, ns, not significant; ***p<0.001; ****p<0.0001.

We then determined the expression of CD27 on the surface of γδ T cells. In the cervical LNs of neutropenic mice, 78.6% of γδ T cells were CD27^-^ cells, significantly higher than the corresponding value (23.2%) in control mice (*p*<0.001) (**Figures 3A, B**), confirming the transcriptomic analysis findings at the protein level. Next, we investigated whether the accumulated γδ T cells in neutropenic mice produced IL-17A. Single cells isolated from the cervical LNs of neutropenic and control mice were cultured in the presence or absence of PMA and ionomycin. As shown in **Figures 3C, D**, under the stimulation of PMA and ionomycin, 67.1% of γδ T cells from neutropenic mice produced IL-17A, a significantly higher percentage than that in γδ T cells from control mice (26.7%) (p<0.0001). Given that γδ T cells consist of two major subtypes—IL-17A-producing and IFN-γ-producing cells (16), we also investigated whether neutrophil deficiency affected IFN-γ-producing γδ T cells. However, no significant differences were observed in the frequency of IFN-γ-producing γδ T cells between neutropenic mice and littermate controls (**Supplementary Figure 4**).

### *Neutrophils regulate the IL-17A producing γδT cells in vitro* in a contact-dependent manner

Since IL-17A is an essential cytokine involved in recruiting neutrophils into peripheral tissues (17), the accumulation of IL-17A producing γδ T cells in neutropenic mice led us to hypothesize that neutrophils regulate IL-17A producing γδ T cells and that this feedback loop might be crucial for maintaining homeostasis.

**Figure 4:**
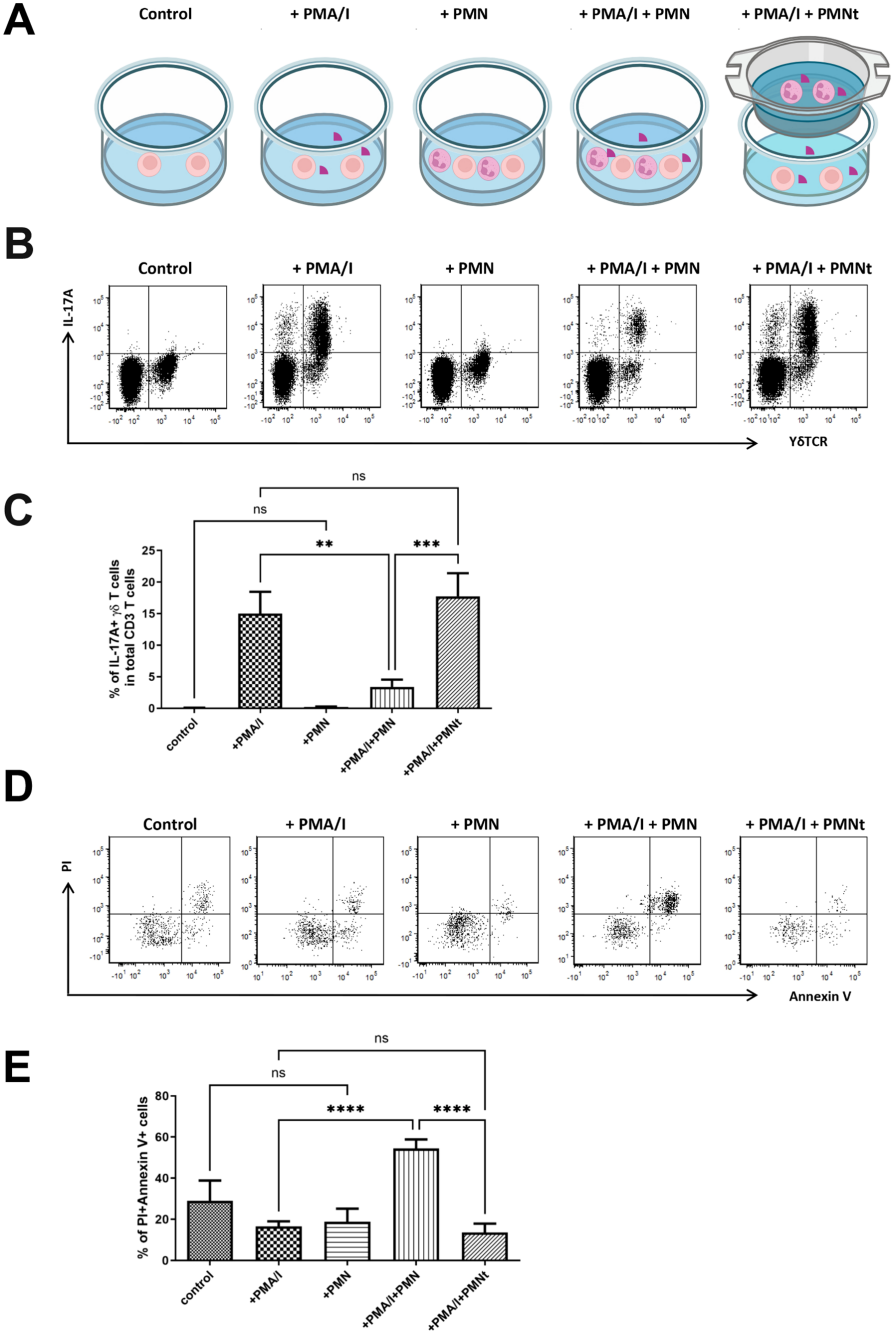
Neutrophils regulate IL-17A production and cell death of γδT cells *in vitro*. **(A)** Schematic diagram of the detailed experimental conditions. Single-cell suspensions were prepared from the cervical LNs of neutropenic mice (n=6) and cultured for 5 hours with or without stimulation of phorbol myristate acetate and calcium ionophore (PMA/I), in presence or absence of neutrophils (PMN) isolated from littermate control mice at 1:1 ratio, or presence of neutrophils in a transwell co-culture manner (PMNt). In the transwell co-culture system, which employed transwell inserts, single-cell suspensions prepared from the cervical LNs were seeded into the wells of the culture plate, while neutrophils were seeded onto the transwell inserts. After the culture, cells were collected and stained with surface markers to define γδT cells and with anti-IL-17A IgG to determine the expression of IL-17A. **(B)** Representative samples show the expression of IL-17A in gated living CD3 T cells, where living γδT cells are further defined as γδTCR positive cells. Quantitative values of percentage of IL-17A^+^ γδT cells in CD3 cells are shown in **(C)** Cell death was evaluated by staining the cultures cells with Annexin V and propidium iodide (PI). **(D)** Representative samples show the cell death of total γδT cells which are defined as CD3^+^γδT^+^ cells. Quantitative values of percentage of Annxin V^+^PI^+^ γδT cells (dead cells) are shown in **(E)** Data are presented as mean ± SEM, ns, not significant; **p<0.01; ***p<0.001; ****p<0.0001.

To verify this hypothesis, we isolated single cells from the cervical LNs of neutropenic mice and co-cultured them with *in vitro* differentiated neutrophils from the bone marrow of littermate control mice in the presence or absence of PMA and ionomycin (**Figure 4A**). Under stimulation with PMA and ionomycin, the major IL-17A producing cells in the cervical LNs of neutropenic mice were CD3^+^ T cells, and the predominant IL-17A^+^ T cells were γδ T cells (data not shown). Notably, when the cells were stimulated with PMA and ionomycin in the presence of neutrophils at a 1:1 ratio, the percentage of IL-17A^+^ γδ T cells among total CD3^+^ T cells decreased drastically from 15.1% to 3.93%, suggesting that neutrophils regulate IL-17A producing γδ T cells. However, when neutrophils were added to a transwell, the regulatory effect of neutrophils on IL-17A producing γδ T cells disappeared (**Figures 4B, C**), indicating that this regulation is contact-dependent.

Based on this observation, we hypothesized that neutrophils induce the death of IL-17A producing γδ T cells. We then set out to verify this hypothesis by determining cell death using Annexin V/Propidium iodide (PI) staining, which distinguishes living cells (Annexin V^-^PI^-^), early apoptotic cells (Annexin V^+^PI^-^) and dead cells (Annexin V^+^PI^+^). As shown in **Figures 4D, E**, in the presence of PMA and ionomycin stimulation, neutrophils significantly increased the percentage of dead γδ T cells from 16.5% to 54.5% (p < 0.0001). However, this effect on γδ T cell death was not observed in the absence of PMA and ionomycin, suggesting that the effect is activation-dependent. Furthermore, the effect also disappeared when neutrophils were placed in a transwell (**Figures 4D, E**), demonstrating that the effect is contact-dependent.

**Figure 5:**
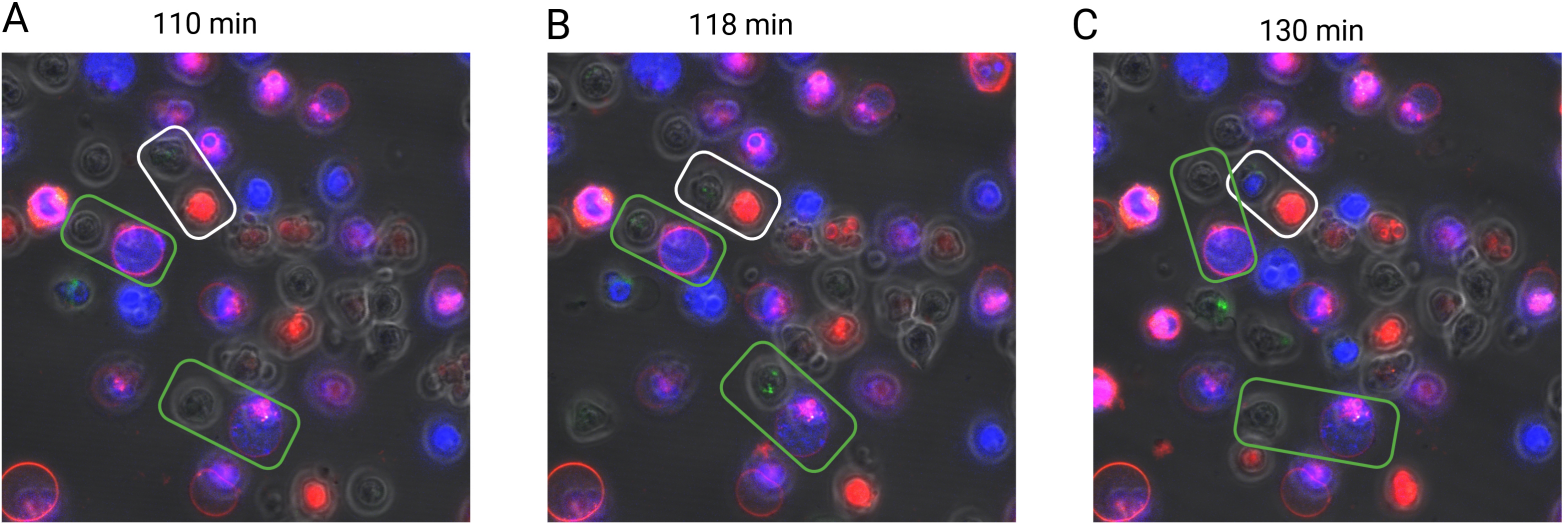
Contact-dependent induction of γδT cell death by living neutrophils. Sorted murine γδT cells (green) from neutropenic mice were co-cultured with *in vitro*-differentiated neutrophils (red) in the presence of PMA (100 ng/ml) for three hours. Dead cells are indicated by DAPI-positive staining (blue). The interaction between γδT cells and neutrophils was monitored using a confocal microscope every 2.5 minutes. Rectangular boxes highlight γδT cells and neutrophils in direct contact, with white boxes indicating interactions with living neutrophils and green boxes indicating interactions with dead neutrophils. Representative images are shown at three distinct time points corresponding to different interaction stages: before contact [110 min, **(A)**], during contact [118 min, **(B)**], and after contact [137 min, **(C)**].

To further validate the contact-dependent effect, γδ T cells sorted from the cervical lymph nodes and spleens of neutropenic mice were co-cultured with bone marrow-derived wild-type neutrophils labeled using Vybrant cell labeling solutions at a 1:1 ratio. The cells were cultured in a chamber at 37°C for 200 minutes in the presence of PMA and calcium ionophore, with DAPI staining used to identify dead cells. Throughout the culture period, a series of images was captured to generate a time-lapse video, allowing visualization of cell interactions (**Supplementary Video 1**). As shown in the representative images in **Figure 5**, γδ T cells underwent cell death within minutes of direct contact with living neutrophils. In contrast, interactions with dead neutrophils did not induce γδ T cell death, highlighting the requirement for live neutrophils in this contact-dependent effect.

## Discussion

In this study, we present novel data on the regulatory role of neutrophils in the immune system. Our data indicate that *in vivo* constitutional neutrophil deficiency leads to the accumulation of IL-17A producing γδ T cells in mice. Furthermore, neutrophils regulate IL-17A producing γδ T cells *in vitro*, suggesting their regulatory role under physiological conditions.

Interestingly, neutropenic mice are characterized by the accumulation of γδ T cells, but not other cell types. This finding suggests that although neutrophils are capable of regulating various immune cell types (18), γδ T cells are the primary targets of neutrophil regulation under physiological conditions. This is consistent with previous observations in humans, where Bank et al. reported that patients with chronic neutropenia exhibit proliferation of γδ T cells (19). A possible explanation is that under physiological conditions, innate immunity tends to be activated rather than adaptive immunity. Consequently, γδ T cells rather than CD4+ T cells are activated and thus need regulation by neutrophils. It has been reported that γδ T cells can be categorized into two groups based on their cytokine profile: IL-17A- and IFN-γ-producing γδ T cells (15). The accumulated γδ T cells in neutropenic mice are predominantly IL-17A-producing cells, which are involved in recruiting neutrophils to peripheral tissues. This suggests a crosstalk between neutrophils and IL-17A producing γδ T cells under physiological conditions.

Our *in vitro* results show that neutrophils inhibit IL-17A producing γδ T cells in the presence of PMA/ionomycin as activators, indicating that the neutrophil-mediated inhibition of γδ T cell death is activation-dependent. Furthermore, the inhibitory effect of neutrophils on activated IL-17A producing γδ T cells disappeared when neutrophils were placed in a transwell, suggesting that the effect is cell contact-dependent. This notion is further supported by the observation that γδ T cells sorted from neutropenic mice rapidly underwent cell death upon direct contact with living neutrophils. This aligns with findings from previous studies by Sabbione et al. and Costa et al., which reported that neutrophils inhibit γδ T cell functions in a cell contact-dependent manner (6, 9). Unlike previous studies that suggest neutrophils inhibit γδ T cell proliferation (6, 9), our study suggests that neutrophils induce cell death in activated IL-17A producing γδ T cells. This disparity might be due to differences in *in vitro* experimental settings. In our study, γδ T cells were stimulated with PMA and ionomycin and co-cultured with neutrophils for 5 hours, whereas in the previous studies, γδ T cells were stimulated with CD3/CD28 or HMBPP and co-cultured with neutrophils for 3 or 4 days (6, 9). Nevertheless, depending the experimental conditions, neutrophils are capable of both inhibiting proliferation and inducing cell death of IL-17 producing γδ T cells. Given the contact-dependent effect of neutrophils on γδ T cells, understanding the underlying molecular mechanisms is of significant interest. Costa et al. have suggested that neutrophil-mediated inhibition of γδ T cell function occurs through the generation of reactive oxygen species (ROS), a process dependent on the kinase Syk (9). However, whether this ROS-Syk axis also plays a role in neutrophil-induced cell death of IL-17-producing γδ T cells remains to be investigated.

The negative regulation of IL-17A producing γδ T cells by neutrophils may be relevant for *in vivo* regulation of immune responses. γδ T cells are a unique T cell subpopulation that are rare in secondary lymphoid organs. By rapidly producing cytokines, γδ T cells play an essential role in immune surveillance and in steady-state tissue physiology (20). Under specific pathogen-free housing condition, neutropenic mice, but not their littermate controls, show accumulation of IL-17A producing γδ T cells primarily in the cervical LNs. This intriguing phenomenon suggests that certain physiological stimuli associated with the oral environment may activate IL-17A-producing γδ T cells. Subsequently, IL-17A secreted from activated γδ T cells exerts its effects on tissue epithelial cells, inducing the production of chemoattractant that facilitate the recruitment of neutrophils. In consequence, neutrophils are recruited from the bloodstream to the affected tissue, where they engage in the eradication of the non-pathogenic stimuli. Upon successful clearance of the stimuli, neutrophils transition to a regulatory role, whereby they induce apoptosis in the activated IL-17A producing γδ T cells, thereby tempering the immune response and restoring homeostasis. In the case of neutrophil deficiency, activated IL-17A producing γδ T cells cannot be eliminated by neutrophils, leading to their accumulation in the cervical LNs. However, this hypothetical model requires experimental validation.

This study has two major limitations. First, the transcriptome analysis was performed using bulk γδ T cells, which precludes the examination of specific differences in IL-17A-producing γδ T cells between neutropenic mice and their littermate controls. Second, although the present study demonstrates that neutrophils regulate IL-17A-producing γδ T cells in a cell-contact-dependent manner, it does not provide evidence of the molecular mechanisms underlying this regulation.

In summary, our study demonstrates that neutrophils regulate the cell death of IL-17A producing γδ T cells both *in vivo* and *in vitro*. Given that IL-17A indirectly promotes the recruitment of neutrophils (21), this finding underscores the crosstalk between neutrophils and IL-17A producing γδ T cells under physiological conditions.

## Data Availability

The datasets presented in this study can be found in online repositories. The names of the repository/repositories and accession number(s) can be found in the article/**Supplementary Material**.
