# Supplementary material for: Neutrophils negatively control IL-17A-producing γδ T cell frequencies in a contact-dependent manner under physiological conditions
Source: Front Immunol. 2025 Mar 20;16:1542191. doi: 10.3389/fimmu.2025.1542191 (PMC11965643; doi:10.3389/fimmu.2025.1542191)
Supplement: Supplementary file 3 [file DataSheet1.pdf]

Supplementary figures

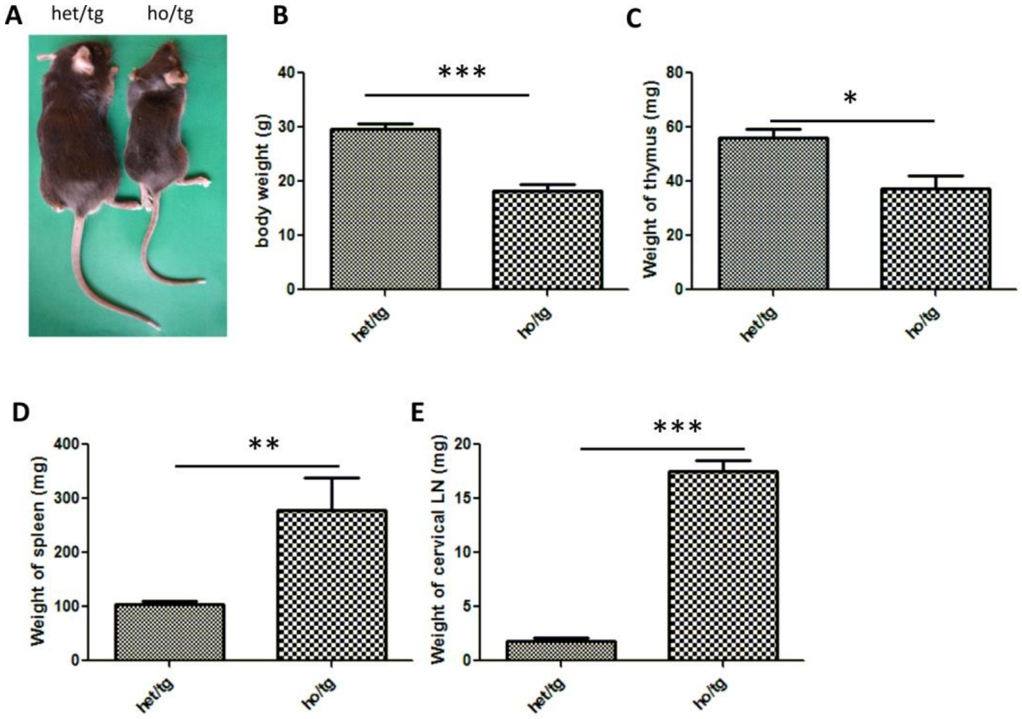

Figure S1. Weight of body and lymph organs of neutropenic and control mice. Male mice at the age of 10-12 weeks old were determined for weight of body and lympho organs. A. Representative picture of *Mcl-1*$^{f/f}$; LysM^{Cre} (ho/tg) neutropenic mice and their littermate *Mcl-1*$^{f/wt}$; LysM^{Cre} (het/tg) control mice. Quantitative values of weight of body (B), weight of thymus (C), spleen (D) and cervical LN (E) of neutropenic mice (n=6) and control mice (n=6). Data are presented as mean±SEM, statistical analysis was performed using Graphpad Prism5 software (***, p<0.001).

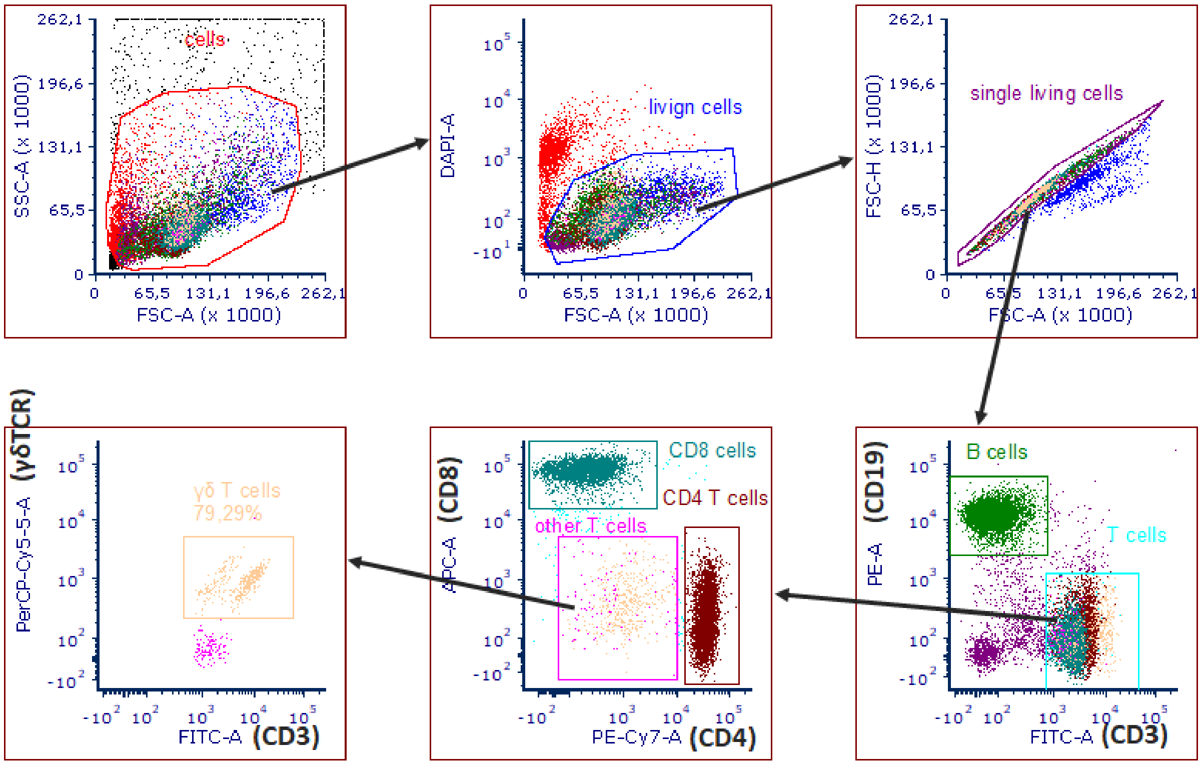

Figure S2. Gating strategy of defining subpopulation of lymphocytes. Single-cell suspensions were prepared from the cervical lymph nodes and stained with FITC-rat anti-mouse CD3, PerCP/Cy5.5 anti-mouse TCR γδ, PE-anti-mouse CD19, PE/Cy7-anti-mouse CD4 and APC-anti-mouse CD8a, and detected using flow cytometric device BD^{TM} LSRII. Data were analyzed with the FCSExpress software.

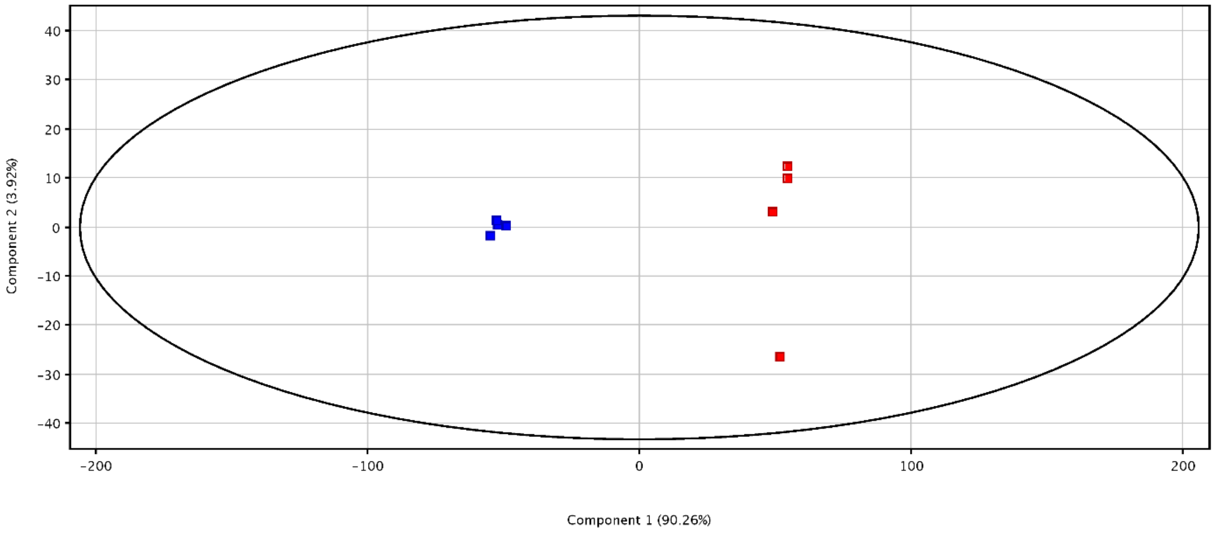

Figure S3. Principal component analysis of the samples of γδT cells from neutropenic (ho/tg) and control (het/tg) mice.

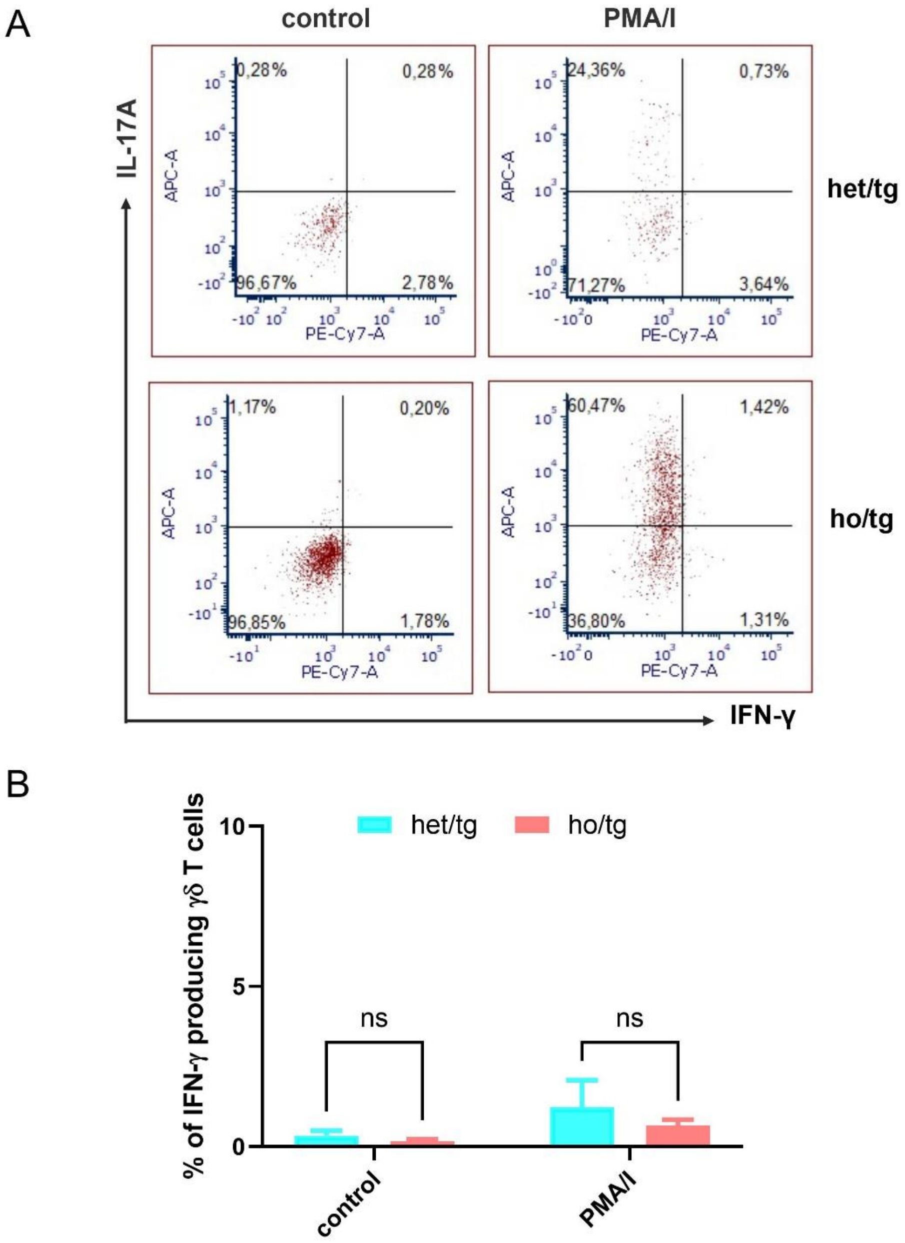

Figure S4. IFN-γ production in γδ T cells from neutropenic mice and littermate controls. Single-cell suspensions were prepared from the cervical lymph nodes of neutropenic mice (ho/tg, n=3) and littermate controls (het/tg, n=3) and cultured either in the absence (control) or presence of phorbol myristate acetate and calcium ionophore (PMA/I). Cells were subsequently analyzed by intracellular staining using Alexa Fluor 647-conjugated anti-mouse IL-17A and PE/Cy7-conjugated anti-mouse IFN-γ. (A) Representative dot plot showing IL-17A and IFN-γ expression in γδ T cells from neutropenic mice and littermate controls. The numbers in the upper left and lower right quadrants indicate the percentages of IL-17A^{+} and IFN-γ^{+} cells, respectively. (B) Quantification of the percentage of IFN-γ-producing γδ T cells is shown in panel D. Data are presented as mean ± SEM, with *ns* indicating no significant difference.
